# Assessing Psychiatric Comorbidity and Pharmacologic Treatment Patterns Among Patients With Neurofibromatosis Type 1

**DOI:** 10.7759/cureus.20244

**Published:** 2021-12-07

**Authors:** Alexander C Houpt, Shaina E Schwartz, Robert A Coover

**Affiliations:** 1 Clinical Sciences, High Point University Fred Wilson School of Pharmacy, High Point, USA; 2 Basic Pharmaceutical Sciences, High Point University Fred Wilson School of Pharmacy, High Point, USA

**Keywords:** antipsychotic, antidepressant, treatment, psychopharmacology, attention-deficit/hyperactivity disorder, anxiety, depression, neurofibromatosis type 1

## Abstract

Background and objective

Neurofibromatosis 1 (NF1) is a genetic disorder that is accompanied by psychiatric comorbidities such as depression, anxiety, and attention-deficit hyperactivity disorder (ADHD) in more than half of the patients. However, there are limited data describing optimal treatment strategies for these conditions. This study aimed to address that gap in understanding and explore the neurobiological basis of psychiatric comorbidities in NF1.

Materials and methods

A retrospective cohort study was conducted among NF1 patients with a comorbid diagnosis of depression, anxiety, and/or ADHD. These disease states were chosen based on their relatively high reported prevalence in NF1 and shared pathophysiological mechanisms via monoaminergic dysfunction. Information regarding demographics, psychotherapeutic medication use, and clinical outcomes was gathered from electronic medical records. Relationships between patient- and medication-related factors and outcome measures were assessed using statistical analysis.

Results

The study population (n = 82) consisted of NF1 patients with a comorbid diagnosis of depression (76.8%), anxiety (53.7%), and/or ADHD (23.2%). The use of second-generation antipsychotic agent augmentation therapy or hydroxyzine monotherapy was associated with significantly more behavioral health (BH)-related emergency department (ED) visits, admissions, and inpatient days in the study population. Conversely, the use of bupropion augmentation therapy, buspirone augmentation therapy, and stimulants was associated with improved clinical outcomes, though these results were not statistically significant.

Conclusions

Based on our findings in this real-world study setting, patients with NF1 and psychiatric comorbidities appear to experience significant benefits from medications that enhance dopaminergic neurotransmission (e.g., bupropion, stimulants) when compared to drugs that oppose it (e.g., second-generation antipsychotics).

## Introduction

Neurofibromatosis type 1 (NF1) is an autosomal-dominant disorder arising from a mutation in the gene encoding neurofibromin, a tumor suppressor protein involved in activating the RasGAP pathway, which regulates cell growth and differentiation [1,2]. Hallmark symptoms of NF1 involve darkening of the skin and the development of benign neurofibromas that vary in size, shape, and location [1,2]. NF1 is one of the most common neurogenetic disorders, with an estimated incidence rate of one in 3,000 people at birth [3]. Its manifestation begins in childhood and results in a significantly shortened lifespan (median age at death: 59 years, compared to 74 years for the general population in the United States) [4].

Quality of life and psychosocial functioning are notably impaired in patients with NF1 [5,6]. Consequently, this patient population has a higher rate of behavioral and emotional dysfunction [7-9], as well as certain psychiatric disorders [10,11]. The prevalence of autism spectrum disorder is speculated to be as high as 40% in this population [12-14], though a causative link has not yet been established [15]. Attention deficit hyperactivity disorder (ADHD) has also been observed in 38-49% of patients with NF1 [12,16-18] and can cause significant functional impairment [19]. Depression has been reported in an estimated 55% of patients and is particularly problematic due to its association with greater pain intensity in NF1 [6,20,21]. The prevalence of anxiety disorders in NF1 is not well studied but may be present in up to 15% of patients [6,22]. This is in addition to a wide array of cognitive deficits that may accompany the disease. Overall, approximately 80% of children with NF1 will present with a cognitive or behavioral issue [23].

The presence of psychiatric comorbidities in patients with NF1 is likely due in part to the high burden of disease [24], particularly related to skin lesions [25], but may also have a neurobiological basis that is not currently understood [26]. For example, learning deficits in patients with NF1 appear to arise from a different mechanism than ADHD in patients without NF1 [27,28]. The activity of specific neurotransmitter pathways may be implicated. Whole-brain serotonin levels were observed to be elevated in mice models of NF1 [29], and neurofibromin, which is dysregulated in NF1, assists in the activation of serotonin receptor subtype six (5-HT6) [30]. With a decrease in the levels of neurofibromin, there is a concomitant lack of stimulation at the receptor resulting in decreased cyclic adenosine monophosphate (cAMP) and cAMP-responsive element-binding protein (CREB) levels, which are important in the regulation of cell survival, proliferation, and differentiation [30,31]. Antidepressants modulate the signaling pathways of monoamine neurotransmitters such as serotonin and have been studied with regard to the treatment of psychiatric symptoms in NF1. The use of imipramine and fluoxetine has been shown to increase neurogenesis and improve behavioral symptoms in NF1 mice [32]. These compounds directly stimulate the serotonin 5-HT receptor and may correct dysregulated signaling. Interestingly, the serotonin transporter gene SLC6A4 is susceptible to deletion in NF1 [33] but does not appear to be associated with depression in this population [34].

Evidence also suggests that animal models with NF1 have decreased levels of dopamine, though post-synaptic dopamine receptor expression remains unchanged [31,35,36]. Dopamine is important in the long-term potentiation of neurons, a process by which synapses are strengthened to aid in learning and memory formation [37]. The administration of either methylphenidate or L-dopa has been demonstrated to normalize dopamine levels, leading to an increase in attention-related and exploratory behavior in mice with NF1 [35,38]. This association is even stronger in a Drosophila NF1 knockdown, which displays hyperactivity that is ameliorated with methylphenidate [39]. ADHD has been previously linked to genes involved in dopaminergic neurotransmission as well as NF1 [40]. One hypothesis for the biological basis of cognitive dysfunction in NF1 implicates altered dopamine receptor binding [41]. Indeed, NF1 loss in dopamine receptor-expressing spiny neurons has been linked to motor learning delays [42]. The effect of NF1 mutation on learning and memory was linked to neuronal dopamine levels in a dose-dependent manner in animal models [43]. In vivo measurement of dopaminergic neurotransmission in NF1 mice confirms the reduced spontaneous firing [44].

While animal models offer insight into the potential pathogenesis and treatment of psychiatric conditions in patients with NF1, human data is currently scarce, which creates a critical gap in knowledge. This report seeks to provide insight into real-world patterns of medication use in NF1 patients who are diagnosed with psychiatric comorbidities. The primary outcome tested was the impact of medication-related factors on clinical indicators of disease severity [i.e., number of emergency department (ED) visits, admissions, and inpatient days for behavioral health (BH) reasons]. The secondary outcome tested was the impact of patient-related factors (i.e., age, sex, psychiatric diagnosis) on these indicators.

## Materials and methods

This retrospective descriptive analysis was conducted at an 80-bed non-profit BH hospital located in the southeastern United States. Patients were eligible for inclusion if they had an encounter through 1/1/2020 during which a diagnosis of NF1 [International Classification of Diseases (ICD) codes Q85.01 or 237.71] was applied. Patients were excluded from the study if they did not have a comorbid diagnosis of depression (F33), anxiety (F41), or ADHD (F90) on the problem list from the indexed encounter. This study was granted “exempt” status (rule #4) by the Institutional Review Boards at the participating hospital and university in October 2019.

Medical records were reviewed to determine the number of BH ED visits and admissions through 7/1/2020 for each eligible patient. Other information gathered included length of stay and any available treatment-related information (i.e., medication name, dose, duration, and augmentation). Medications were classified as monotherapy (if used alone) or augmentation (if used in combination with another medication indicated to treat the same condition).

Spearman’s rho test was used to calculate non-parametric correlations between continuous patient- and medication-related factors and clinical outcomes (i.e. BH ED visits, BH admissions, BH inpatient days), while Mann-Whitney U test was used to assess this relationship for categorical variables. All statistical analysis was performed using IBM® SPSS Statistics version 26 (IBM, Armonk, NY).

## Results

The study population of patients with NF1 and a comorbid psychiatric diagnosis (n = 82) had a mean age of 44.5 years [range: 6-87 years, standard deviation (SD): 21.5 years] and were mostly female (69.5%) (Table 1). The majority of the patients had a comorbid psychiatric diagnosis of depression (76.8%) or anxiety (53.7%), with 31 (37.8%) having multiple diagnoses. A smaller portion of patients (23.2%) were diagnosed with ADHD. At least one ED visit was noted on 16 patient charts (19.5%) and at least one BH admission was noted on 17 charts (20.7%). Twelve patients (14.6%) had at least one ED visit and BH admission during the study period.

**Table 1:** Baseline characteristics of the study population ADHD: attention-deficit hyperactivity disorder; BH: behavioral health; ED: emergency department; SGA: second-generation antipsychotic; SNRI: serotonin-norepinephrine reuptake inhibitor; SSRI: selective serotonin reuptake inhibitor; TCA: tricyclic antidepressant

Characteristic	Values (n = 82)
Mean age, years	44.5
Sex, n (%)	
Male	25 (30.5%)
Female	57 (69.5%)
Diagnosis of depression, n (%)	63 (76.8%)
Diagnosis of anxiety, n (%)	44 (53.7%)
Diagnosis of ADHD, n (%)	19 (23.2%)
History of BH ED visit, n (%)	16 (19.5%)
Total BH ED visits	30
History of BH admission, n (%)	17 (20.7%)
Total BH admissions	45
Total BH inpatient days	207
Antidepressant use, n (%)	54 (65.9%)
Total SSRI monotherapy	57
Total SNRI monotherapy	17
Total TCA monotherapy	1
Total bupropion monotherapy	7
Total SGA augmentation	10
Total bupropion augmentation	4
Anxiolytic use, n (%)	29 (35.4%)
Total benzodiazepine monotherapy	24
Total hydroxyzine monotherapy	8
Total buspirone monotherapy	5
Total hydroxyzine augmentation	4
Stimulant use, n (%)	10 (12.2%)
Non-stimulant use, n (%)	2 (2.4%)
SGA monotherapy use, n (%)	3 (3.7%)

Antidepressant therapy [i.e., selective serotonin reuptake inhibitors (SSRIs), serotonin-norepinephrine reuptake inhibitors (SNRIs), tricyclic antidepressants (TCAs)] was utilized in 54 (65.9%) patients (65.9%), with many having a history of being prescribed multiple agents (Table 1). Anxiolytics therapy (i.e., benzodiazepines, buspirone, hydroxyzine) was utilized in 29 patients (35.4%). SSRIs were the most common agents prescribed (57 times) followed by benzodiazepines (24 times). Stimulant therapy was used to treat 10 patients (12.2%), while 17 patients (20.7%) in the study population were not being treated with any psychotherapeutic medications.

Age had a significant negative correlation with all clinical outcomes assessed, while the number of SSRIs and the total number of antidepressants positively correlated with BH admissions and inpatient days (Table 2). Similarly, a diagnosis of depression and the use of antidepressants were associated with a significantly higher number of BH admissions and inpatient days (Table 3). The use of second-generation antipsychotic (SGA) augmentation therapy and hydroxyzine monotherapy also emerged as significant positive predictors for all clinical outcomes investigated (Table 3).

**Table 2:** Patient characteristics as predictors for clinical outcomes based on Spearman’s rho test *Denotes statistical significance (p: <0.05) Continuous variables are reported as correlation coefficient (Spearman’s rho) BH: behavioral health; ED: emergency department; SGA: second-generation antipsychotic; SNRI: serotonin-norepinephrine reuptake inhibitor; SSRI: selective serotonin reuptake inhibitor; TCA: tricyclic antidepressant

Variables	BH ED visits	BH admissions	BH inpatient days
Age	r_s _= -0.284* (p = 0.010)	r_s _= -0.221* (p = 0.046)	r_s _= -0.253* (p = 0.022)
Number of SSRIs	r_s _= 0.217 (p = 0.050)	r_s _= 0.354* (p = 0.001)	r_s _= 0.371* (p = 0.001)
Number of SNRIs	r_s _= 0.160 (p = 0.152)	r_s _= 0.159 (p = 0.153)	r_s _= 0.132 (p = 0.236)
Number of TCAs	r_s _= -0.054 (p = 0.627)	r_s _= -0.056 (p = 0.615)	r_s _= -0.050 (p = 0.655)
Number of total antidepressants	r_s _= 0.195 (p = 0.079)	r_s _= 0.267* (p = 0.015)	r_s _= 0.272* (p = 0.013)
Number of benzodiazepines	r_s _= -0.033 (p = 0.771)	r_s _= -0.039 (p = 0.725)	r_s _= -0.051 (p = 0.652)
Number of stimulants	r_s _= 0.104 (p = 0.351)	r_s _= -0.079 (p = 0.480)	r_s _= -0.047 (p = 0.677)
Number of non-stimulants	r_s _= 0.150 (p = 0.178)	r_s _= 0.132 (p = 0.236)	r_s _= 0.163 (p = 0.143)

**Table 3:** Patient characteristics as predictors for clinical outcomes based on Mann-Whitney U test *Denotes statistical significance (p: <0.05) Categorical variables are reported as mean rank with test statistics (Mann-Whitney U) ADHD: attention-deficit hyperactivity disorder; BH: behavioral health; ED: emergency department; SGA: second-generation antipsychotic; SNRI: serotonin-norepinephrine reuptake inhibitor; SSRI: selective serotonin reuptake inhibitor; TCA: tricyclic antidepressant

Variables	BH ED visits	BH admissions	BH inpatient days
Sex	Male = 43.14, Female = 40.78, U = 672 (p = 0.550)	Male = 44.24, Female = 40.30, U = 644 (p = 0.330)	Male = 45.48, Female = 39.75, U = 613 (p = 0.126)
Depression	Yes = 42.69, No = 37.55, U = 674 (p = 0.232)	Yes = 44.06, No = 33.00, U = 760* (p = 0.012)	Yes = 43.61, No = 34.50, U = 732* (p = 0.026)
Anxiety	Yes = 40.13, No = 43.09, U = 776 (p = 0.415)	Yes = 39.60, No = 43.70, U = 753 (p = 0.273)	Yes = 40.18, No = 43.03, U = 778 (p = 0.411)
ADHD	Yes = 44.92, No = 40.47, U = 664 (p = 0.301)	Yes = 41.63, No = 41.46, U = 601 (p = 0.969)	Yes = 41.39, No = 41.53, U = 587 (p = 0.973)
Use of antidepressants	Yes = 43.36, No = 37.91, U = 857 (p = 0.154)	Yes = 44.41, No = 35.89, U = 913* (p = 0.030)	Yes = 44.29, No = 36.13, U = 907* (p = 0.035)
Bupropion monotherapy	Yes = 45.79, No = 41.10, U = 293 (p = 0.471)	Yes = 39.79, No = 41.66, U = 251 (p = 0.778)	Yes = 41.29, No = 41.52, U = 261 (p = 0.970)
Bupropion augmentation	Yes = 33.50, No = 41.91, U = 124 (p = 0.512)	Yes = 33.00, No = 41.94, U = 122 (p = 0.486)	Yes = 34.50, No = 41.86, U = 128 (p = 0.568)
SGA monotherapy	Yes = 61.83, No = 40.73, U = 180 (p = 0.139)	Yes = 62.67, No = 40.70, U = 182 (p = 0.125)	Yes = 65.17, No = 40.60, U = 190 (p = 0.081)
SGA augmentation	Yes = 55.30, No = 39.58, U = 498* (p = 0.005)	Yes = 60.15, No = 38.91, U = 547* (p: <0.001)	Yes = 60.30, No = 38.89, U = 548* (p: <0.001)
Use of benzodiazepines	Yes = 40.80, No = 41.76, U = 645 (p = 0.814)	Yes = 40.59, No = 41.83, U = 640 (p = 0.767)	Yes = 40.34, No = 41.93, U = 635 (p = 0.684)
Hydroxyzine monotherapy	Yes = 69.44, No = 38.48, U = 520* (p: <0.001)	Yes = 64.38, No = 39.03, U = 479* (p: <0.001)	Yes = 65.81, No = 38.87, U = 491* (p: <0.001)
Hydroxyzine augmentation	Yes = 44.75, No = 41.33, U = 169 (p = 0.795)	Yes = 54.50, No = 40.83, U = 208 (p = 0.279)	Yes = 56.38, No = 40.74, U = 216 (p = 0.209)
Buspirone monotherapy	Yes = 50.20, No = 40.94, U = 236 (p = 0.416)	Yes = 50.80, No = 40.90, U = 239 (p = 0.384)	Yes = 51.40, No = 40.86, U = 242 (p = 0.353)
Buspirone augmentation	Yes = 33.50, No = 41.60, U = 33 (p = 0.805)	Yes = 33.00, No = 41.60, U = 32 (p = 0.805)	Yes = 34.50, No = 41.59, U = 34 (p = 0.829)
Use of stimulants	Yes = 45.85, No = 40.90, U = 404 (p = 0.372)	Yes = 37.45, No = 42.06, U = 320 (p = 0.417)	Yes = 39.05, No = 41.84, U = 336 (p = 0.596)
Use of non-stimulants	Yes = 56.75, No = 41.12, U = 111 (p = 0.391)	Yes = 55.25, No = 41.16, U = 108 (p = 0.439)	Yes = 57.25, No = 41.11, U = 112 (p = 0.376)

## Discussion

Our examination of treatment patterns in patients with NF1 and a comorbid psychiatric diagnosis has revealed potential links between the neurobiological basis of these conditions and various pharmacological treatment approaches. Specifically, the modulation of dopamine signaling may be implicated based on the findings of the present study. The use of augmentation therapy with SGAs, which block dopamine receptors, was observed to have a negative impact on clinical outcomes in the study population. Conversely, it appears that the use of bupropion augmentation and stimulant medications, which enhance dopamine signaling via reuptake inhibition mechanisms, may be associated with improved clinical outcomes, though these findings were not statistically significant. Buspirone augmentation therapy was also associated with a non-significant reduction in the primary outcome measures, but its effect on dopamine neurotransmission is complicated. Patients who were younger tended to have more ED visits and longer inpatient days, which makes it unlikely that the effects seen were results of psychiatric disease duration and/or treatment experience.

A year-long clinical study of children with NF1 found that the administration of the stimulant medication methylphenidate significantly improved cognitive and academic performance as well as social deficits [17]. Double-blind placebo-controlled crossover trials have demonstrated the efficacy of methylphenidate in reducing ADHD symptoms at four weeks [45] and cognitive symptoms at six weeks [46]. The efficacy of stimulants in patients with NF1 and ADHD has been theorized to relate to the predominance of the combined subtype in this population [47]. Though the present study did not find any significant links between the use of non-stimulant medications and clinical outcomes in patients with NF1, previous research suggests that guanfacine may ameliorate symptoms of ADHD in NF1 mouse models [48]. While the extent to which altered dopamine signaling in patients with NF1 impacts the treatment of psychiatric comorbidities remains unclear, the role of dopamine in the pathophysiology of depression has been previously explored. The dopamine agonist amantadine was studied among a small group of patients with treatment-resistant depression [49], and improvements were observed in both anxiety and depression scores [49], suggesting that dopamine plays a role in depression and anxiety, making it a potential treatment target.

There are several important limitations regarding the outcomes of this observational study. SGAs as augmentation therapy are generally reserved for patients who still experience depression or anxiety symptoms despite monotherapy treatment with a first-line agent. Patients in this study who were treated with SGAs had a higher rate of BH admissions, which may have been due to the severity of the patient’s psychiatric condition rather than the antipsychotic medication itself. The association of a depression diagnosis, use of antidepressants, number of SSRIs, and total antidepressants with poorer clinical outcomes in the study population support the presence of a more acute subpopulation. It is not clear why hydroxyzine, an antihistamine agent often prescribed for its anxiolytic properties, was also linked to worse outcomes when used as monotherapy in this study, though its use in this manner is generally not recommended. A small sample size due to the rare nature of NF1 is another important limitation of this study.

While there was an association between the prescription of pro-dopaminergic agents and fewer BH** **ED visits, admissions, and inpatient days in the study population, there is insufficient evidence to definitively state that better outcomes were a direct result of the use of these medications. Future research into the relationship between dopamine and NF1 is needed to better define the role of dopamine-modulating agents in the treatment of psychiatric comorbidities in these patients.

## Conclusions

Psychiatric comorbidities (depression, anxiety, and/or ADHD) are frequently observed in patients with NF1. Using BH ED visits, admissions, and inpatients days as clinical indicators, dopamine-blocking therapy with antipsychotic medications was found to be associated with worse outcomes, while dopamine-enhancing therapy with bupropion or stimulant medications was associated with improved outcomes. These findings suggest further areas of research to optimize the treatment of psychiatric comorbidities in patients with NF1.
